# The microbiota-gut-brain axis in stress and depression

**DOI:** 10.3389/fnins.2023.1151478

**Published:** 2023-04-14

**Authors:** Hwei-Ee Tan

**Affiliations:** Lee Kong Chian School of Medicine, Nanyang Technological University, Singapore, Singapore

**Keywords:** stress, perception, brain-gut axis, microbiome, microbiota-gut-brain connection, depression, mindfulness, host–microbe interactions

## Abstract

Humans and animals are evolved to have instinctive physiological responses to threats. The perception of threat by the brain triggers a multitude of changes across the brain and body. A large body of research have demonstrated that our hardwired survival instinct, the stress response, can become maladaptive and promote major depressive disorders and other neuropsychiatric impairments. However, gaps in our understanding of how chronic stress contributes to depression and mental disorders suggest that we also need to consider factors beyond the biology of the host. The unravelling of the structure and function of microorganisms that humans and animals are host to have driven a paradigm shift in understanding the individual as a collective network composed of the host plus microbes. Well over 90% of bacteria in the body reside in the large intestines, and these microbes in the lower gut function almost like an organ in the body in the way it interacts with the host. Importantly, bidirectional interactions between the gut microbiota and the brain (i.e., the two-way microbiota-gut-brain axis) have been implicated in the pathophysiology of mental disorders including depression. Here, in summarizing the emerging literature, we envisage that further research particularly on the efferent brain-gut-microbiota axis will uncover transformative links in the biology of stress and depression.

## 1. Introduction

Living beings survive in a changing environment by responding adaptively to stressors that threaten our well-being (Cannon, 1915). Such threat signals may come from external sources, for example a startling sound, or physical abuse, as well as internal sources, such as a medical illness or inadequate sleep (Selye, 1956). All stressors are ultimately perceived in the brain *via* our exteroceptive and interoceptive senses as stimuli that signal a potential destabilisation of our homeostatic wellbeing, and such input into the brain will innately trigger the stress response (McEwen, 2007; Sterling, 2012). The stress response is in essence an orchestrated change in physiological and neural states that is most intuitively characterized by heightened alertness, and is remarkably conserved across all vertebrate animals (Romero and Butler, 2007; Lovejoy et al., 2014).

The transition to a stressed physiological state throughout the body is coordinated by the brain through 2 principal pathways: a rapid autonomic neural pathway from the brain to the adrenal medulla and other organs through efferent sympathetic nerves, and a slower neuroendocrine pathway from the hypothalamus in the brain to the adrenal cortex *via* the pituitary gland (Romero and Butler, 2007; Samuels and Szabadi, 2008; Lupien et al., 2009; Ulrich-Lai and Herman, 2009; Herman et al., 2016; Martin et al., 2018). Activation of noradrenergic efferent neurons in the locus coeruleus brain region engages the sympathetic pathway of the autonomic nervous system within seconds of perceiving a stressful stimulus, driving the release of catecholamines such as adrenaline and noradrenaline which activates the classical ‘fight or flight’ response, including increased heart rate and contraction force, bronchodilation and increased respiration rate, and energy mobilisation from liver stores. In parallel, corticotropin-releasing hormone secreting neurons in the paraventricular hypothalamus initiate the neuroendocrine hypothalamic–pituitary–adrenal (HPA) pathway, releasing the glucocorticoid cortisol into circulation in the subsequent tens of minutes, which suppresses immune and digestive functions and modifies metabolic pathways for positive energy balance. By shutting down non-essential systems and providing a boost of energy, this hardwired physiological stress response enables humans and other animals to react quickly to potentially life-threatening stimulus.

In addition to flipping the “survival mode” switches throughout the body, the perception of stressful stimuli in the brain also modulates neural circuits within the central nervous system that dictate mental states and behaviour. For example, although the locus coeruleus controls peripheral autonomic effects as described above, the stimulation of central noradrenergic projections from the locus coeruleus robustly induces a state of arousal in the brain (Samuels and Szabadi, 2008; Zerbi et al., 2019). Stressful stimuli also engage various brain regions including the amygdala, which tunes emotional states and influences anxiety, sociability, aggressiveness (McEwen, 2007; Lupien et al., 2009; Myers et al., 2017).

## 2. From stress to depression

While the hardwired stress response in humans and animals must have been indispensable in safeguarding our homeostatic wellbeing over the course of evolution, our instinctive stress response becomes maladaptive when repeatedly or continuously exposed to stressors over long time periods (an experience that had surfaced only from the most recent ~0.02% of human history; Selye, 1956; Lupien et al., 2009; Lovejoy et al., 2014; Pearson, 2021). The experience of severe or chronic stress is the major driver of neuropsychiatric illnesses such as clinical depression, an increasingly common mental disorder characterized most saliently by low mood and a loss of interest among other symptoms (Kessler, 1997; Hammen, 2005; Casey et al., 2013). For instance, in a co-twin control study, severe, stressful life events were significantly associated with the onsets of depression even after filtering only for independent stressors (such as assault, housing woes, bereavement), suggesting a substantial causal relationship (Kendler et al., 1999). In addition, persistent stressors spanning a long time period also precipitates major depressive disorders (Melchior et al., 2007; Steinhardt et al., 2011). Moreover, unpredictable catastrophic disasters (e.g., 2008 financial crisis, 2019 coronavirus pandemic) are linked to an increase in mental disorders including depression at a population scale (Burgard et al., 2009; Mucci et al., 2016; Forbes and Krueger, 2019; Salari et al., 2020; World Health Organization, 2022). Taken together, human studies concur that stress is significantly associated with major depressive disorders.

Furthermore, strong evidence that stress indeed causes depression has emerged from a series of investigations whereby non-human animal models are experimentally exposed to various stressors. In rodents, depriving the pups from maternal care for a few hours a day within the first 2–3 weeks since birth increases behavioural measures of anxiety and depression in adulthood, just like early childhood adversity would in humans (Levine and Wiener, 1988; Bremner and Vermetten, 2001; Franklin et al., 2010). Physical restraint is another potent stressor as it leaves animals vulnerable to threats and deprived of homeostatic needs such as obtaining food and water (Narvaez et al., 1993; Poleszak et al., 2006). After a single episode of prolonged 24-h restraint, perhaps analogous to humans being trapped in rubble, adult mice display lasting depression-like symptoms such as anhedonia and despair even up to 35 days after the restraint event (Chu et al., 2016). Besides maternal separation and physical restraint, other well established deliberate stress regimens include social defeat and chronic mild stress (Kudryavtseva et al., 1991; Willner, 1997; Iñiguez et al., 2014; Monteiro et al., 2015; Harris et al., 2018). Generally, animals subjected to stress paradigms consequently display behaviours that mirror symptoms of depressive disorders, including decreased sucrose preference (anhedonia), increased immobility in forced swim tests (despair), decreased time exploring the brighter side of a two-chambered light–dark box (anxiety), reduced self-grooming (apathy), and decreased interaction with a conspecific (reduced sociability; Porsolt et al., 1977; Cryan et al., 2002; Nollet, 2021).

This condensed overview presents the association and causality of stress in depression, but we must mention that there is huge variability in methods, observations, and interpretations across the expansive literature (Willner, 1997; Jung et al., 2014; Nollet, 2021). Such observed variability – even in laboratory animal models where many factors such as genetics and physical environment can be tightly controlled – indicates that there may be other overlooked contributing factors that link stress to depression.

## 3. Depression’s elusive pathogenesis

Although a complete understanding of how stress transforms into depression remains elusive, it is apparent that peripheral and central factors can contribute to the neural basis of depression.

The cortisol hormone, released *via* the neuroendocrine HPA axis as part of the stress response, is normally under tight self-regulation through a negative feedback loop. Cortisol released in circulation acts on glucocorticoid receptors expressed in the paraventricular hypothalamus to dial down the HPA output, hence inhibiting further release of cortisol by the adrenal gland. However, such feedback inhibition of the HPA axis is weakened in depressed patients, leading to a hyperactive HPA axis and elevated cortisol levels, which have been suggested to be the basis of depression (Pariante and Lightman, 2008; Zhao et al., 2008; Gourley and Taylor, 2009). However, it is unclear what impairs the negative feedback loop involving cortisol and the HPA axis (Pariante and Lightman, 2008; Kling et al., 2009).

In contrast to the general immunosuppressive effect of a normal, brief stress response, a paradoxical inflammatory response triggered by stress may also impair brain circuits in a manner that causes depression. Patients of major depressive disorders present with heightened activation of microglia, which are immune cells that surveil the brain (Perry, 2018; Wang et al., 2022). In experimental systems, chronic social defeat stress also induces microglia activation in the brain (Nie et al., 2018). As such, neuroinflammatory impairment of neural circuits may underlie depression. Yet, it remains puzzling how the experience of a stressor such as social defeat mechanistically transduces biochemical activation of those microglia cells.

Changes in neural circuits underlie mental disorders. Such innate plasticity of brain circuits is not surprising, as the growth or death of neurons and their connections are the defining features of the brain. For example, neurodegeneration in certain brain areas, which can also be surgically induced in animal models, is sufficient to create depression-like phenotypes (Kelly et al., 1997; Harkin et al., 2003; Soudry et al., 2011). Neuronal hyperexcitability in habenular neurons, caused by altered genetic and cellular programmes, also causes depression phenotypes in rodents (Li et al., 2013; Hu et al., 2020; Fan et al., 2023). However, these explanations are incomplete in linking stress to depression: for example, how does experiencing stressors lead to lasting hyperexcitability-associated intracellular programmes in those habenular neurons in the first place?

While our understanding of the neurobiology of stress and depression has progressed remarkably over these years, the seemingly elusive biological mystery is how transient stressor events can permanently alter brain circuits into that of depression. If this puzzle appears unsolvable, could we be missing a piece of the puzzle?

## 4. Microbial factors in depression

Mainstream research has been focused on the biology of the host and largely overlooked the role of the microbiota, which may be the missing major piece that will allow us to assemble a more complete model of how stress causes depression. In an average person, microbes outnumber human cells – at approximately 38 trillion bacteria to 30 trillion human cells (Sender et al., 2016). Our human cells are in an inseparable and mutualistic relationship with our resident microbiota, and therefore it would be prudent to consider both host and microbiota factors in modelling the biology of a person. An assortment of labels has been used in the literature to express this emerging concept, such as characterising an individual as a “superorganism,” a “holobiont,” an “ecosystem,” or having “a microbial organ” (Gordon et al., 2013; Douglas and Werren, 2016; Morar and Bohannan, 2019). While being cognizant that these labels are not equivalent to each other, we suggest these as metaphors to readers new to the microbiome sciences.

If microbiota factors contribute to depression in people, then the microbiome (a genetic census of the microbiota) of depressed patients may display distinct signatures from that of healthy controls. Indeed, by comparing the microbiome of depressed patients versus healthy controls, changes in the abundance of certain bacteria taxa have been reported in many studies (Jiang et al., 2015; McGuinness et al., 2022; Radjabzadeh et al., 2022; Zhong et al., 2022). In agreement with the discovery of metagenomic signatures of depression, metaproteomics and metabolomics studies in mice and men also reveal significant depression-associated differences at the level of microbial-associated peptides and metabolites, the classes of compounds that interface microbe-host interactions (Yu et al., 2017; Chen et al., 2018; Valles-Colomer et al., 2019; Wu et al., 2020).

Remarkably, transferring the microbiota from a depressed donor to a non-depressed recipient results in depression-like phenotypes in the recipient (Zheng et al., 2016; Wang et al., 2020; Knudsen et al., 2021). These preclinical microbiota transplant experiments involving human-to-mouse transfers or mouse-to-mouse transfers substantiate the causality of the gut microbiota in the development of depression.

**Figure 1:**
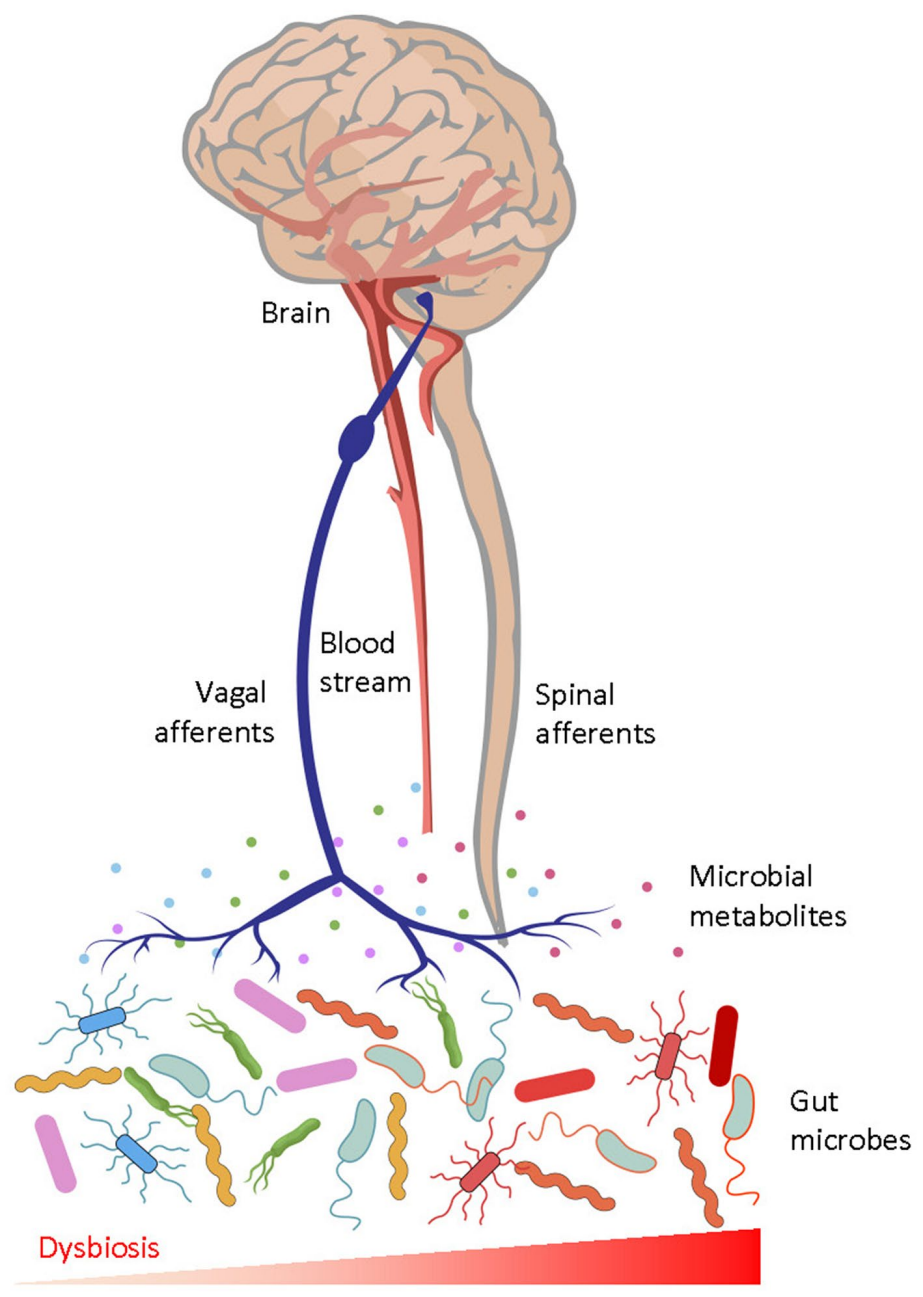
The afferent gut-to-brain axis. Microbial peptides and metabolites interact with peripheral receptors and modulate ascending sensory neurons through vagal and spinal pathways, which then act on neural circuits in the brain. Humoral factors may also enter the brain to modulate function of neurons and glia. Gut dysbiosis, characterised by unfavourable alterations of the gut microbial biochemistry, perturbs physiological gut-brain communication and causes diseases.

Intestinal microbial factors signal *via* the gut-brain axis (in its broadest definition) by activating peripheral sensory afferents or by diffusion into the central nervous system through systemic circulation (Figure 1). For instance, nociceptive sensory neurons that modulate pain and defensive behaviours directly sense bacterial peptides and virulence factors (Chiu et al., 2013). Moreover, sensory nerves also sense microbial products and inflammatory agents *via* intestinal enteroendocrine cells, which express chemoreceptors such as TRPA1 and Olfr558 (Bellono et al., 2017; Ye et al., 2021). Gut-brain vagal afferents engage dopaminergic brain circuits and influence reward-related behaviours, and impairment of gut-brain signals may contribute to anhedonia and despair (Russo and Nestler, 2013; Han et al., 2018; McVey Neufeld et al., 2019; Tan et al., 2020; Ousey et al., 2022). Members of the gut microbiota can also synthesize neurotransmitters, short chain fatty acids, indoles, bile acids, and systemic circulation of these chemicals have been suggested to influence brain circuits and aspects of depression behaviour (Clarke et al., 2013; van de Wouw et al., 2018; Caspani et al., 2019; Needham et al., 2020; Agirman and Hsiao, 2021).

Microbial mechanisms may bridge those puzzling gaps in our understanding that were listed earlier: (i) For instance, microbial engagement of the vagal afferents modulate the responsiveness of the cortisol-HPA axis (La Marca et al., 2011; Chen et al., 2021). (ii) Pattern recognition receptors of the immune system detect a range of microbial factors, and the activation of such receptors mediate microglia maturation and activation (Erny et al., 2017; Keogh et al., 2021). (iii) Short chain fatty acids can enter the central nervous system to regulate histone acetylation and methylation processes. In particular, bacterial butyrate may upregulate gene expression selectively *via* inhibition of histone deacetylases (Wei et al., 2015; Krautkramer et al., 2016).

## 5. Stress imprinting *via* the microbiome

Functionally, the gut microbiota contributes to the persistence of stress effects beyond the “acute” seconds-to-hours period, and allows animals to integrate events temporally over an extended period of several days or weeks. When rats were subjected to restraint stress for an hour a day over 13 days, their caecal microbial metabolites at the end of the chronic stress regimen, or at even up to 3 weeks after, differed significantly from that of non-stressed control rats (Xu et al., 2020). In addition to physical stressors, social stressors also alter the gut microbiome. To model social defeat, an aggressive male mouse can be introduced into a cage with other male mice for 2 h daily over 6 days. In this well-established model, the resident mice experience significant social stress from being repeatedly attacked and defeated daily by the aggressive intruder. Socially defeated mice show a reduction in caecal microbial diversity and richness (Bailey et al., 2011). A follow-up study from the same group revealed that the abundance of *Lactobacillus* group bacteria was reduced after the 1^st^ session of stress, and was further reduced by the 6^th^ session, demonstrating that the gut microbiota can encode the additive effects of stress events over a span of days (Galley et al., 2014).

**Figure 2:**
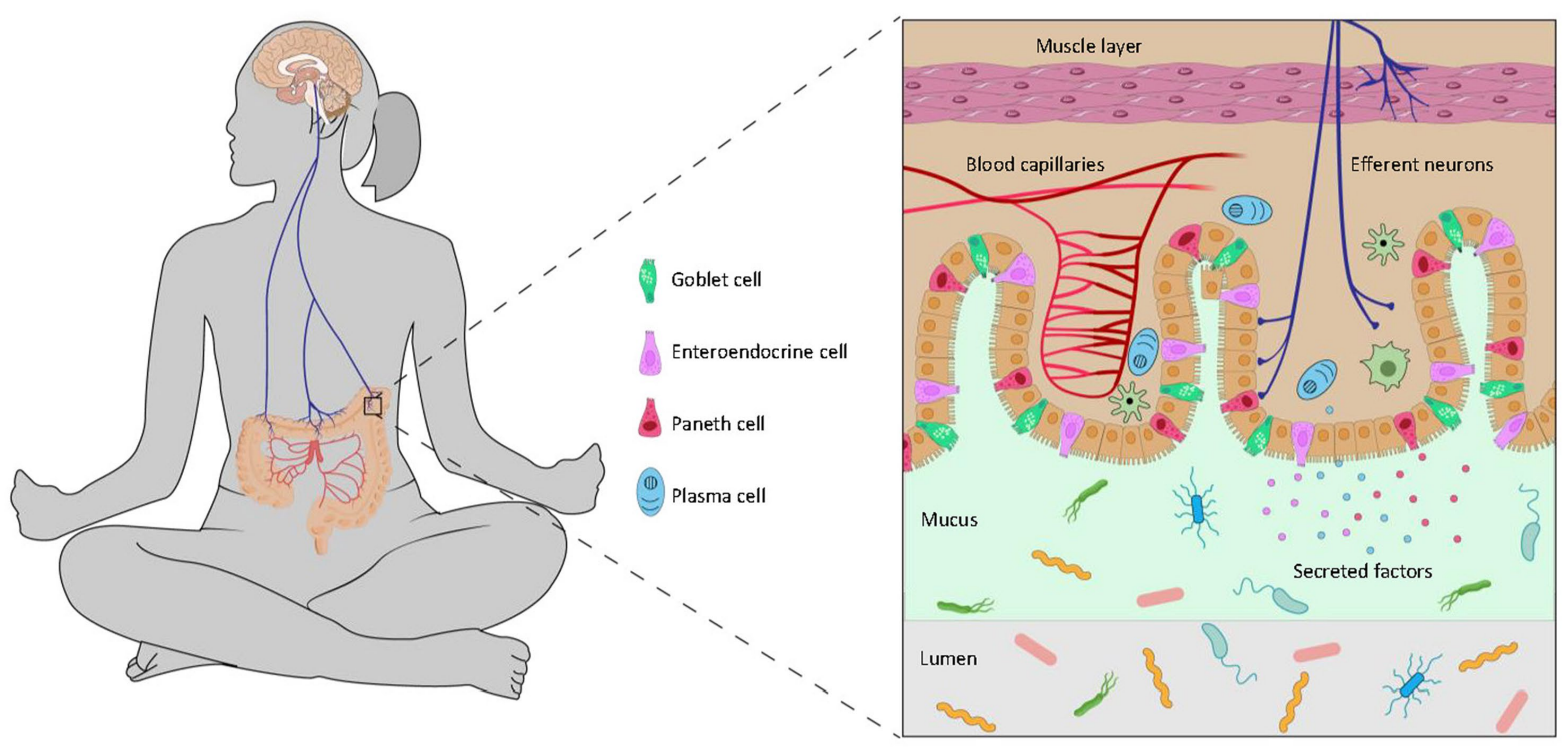
The efferent brain-to-gut axis. The brain controls the gut microbiota through neural and endocrinal pathways. Descending signals to the intestinal epithelium regulate goblet cells which produce mucin, enteroendocrine cells which secrete various peptides, Paneth cells which release anti-microbial compounds, and plasma cells which produce antibodies. These compounds from the host may promote or inhibit the growth of microbes in the gut and influence biochemical pathways of the microbiota.

The central perception of stress directly shapes the microbiota through the efferent brain-gut-microbiota axis (Figure 2).

One pathway is the control of gut peristalsis *via* the autonomic nervous system: extrinsic parasympathetic and sympathetic nerves from the brain innervate a network of intrinsic enteric neurons, which mediate the activity of intramuscular interstitial cells of Cajal and the relaxation and contraction of smooth muscles of the gut. Peristalsis regulates the rate of transit of contents through the intestines. A higher motility of food digesta through the small intestines will result in more unabsorbed nutrients in the lower gut, a factor that will influence the growth of microbes and alter the microbiome composition (Barclay and Turnberg, 1987; Martin et al., 2018). A higher motility through the large intestines will expel luminal microbes more than those in the mucosal or adherent niche, and such disproportionate turnover lead to changes in the microbial community (Aguilera et al., 2013).

The central efferent nerves from the brain also engage secretory cells of the gut either directly or *via* the enteric nervous system. The principal secretory luminal-facing cells in the distal gut include goblet cells, which secrete mucin and maintain the mucus lining, Paneth cells, which secrete anti-microbial compounds, and enteroendocrine cells which secrete a wide range of peptides such as serotonin, peptide tyrosine-tyrosine (PYY) and glucagon-like peptide-1 (Rhee et al., 2009; Solis et al., 2020). These compounds act directly on the microbiota as well as regulate microbe-host signalling (Sommer and Bäckhed, 2016; Allaire et al., 2018).

The brain also regulates host immunity, which is a fundamental regulator of the resident microbiota (Rhee et al., 2009). Placing mice on an elevated platform activates stress-responsive brain regions including the amygdala and paraventricular hypothalamus. These brain regions connect to sympathetic efferents that innervate the spleen to drive humoral immune responses (Zhang et al., 2020). The gut mucosal surface is a highly dynamic interface where the immune defence system keeps the microbial community at bay by preventing bacterial overgrowth or invasion by pathogenic strains, while allowing tolerance to innocuous commensals or harmless foreign antigens. Aberrations of this delicate balance in either direction will alter the microbiota (Sommer and Bäckhed, 2016; Allaire et al., 2018; Agirman and Hsiao, 2021).

In a broader perspective, beyond linking stress to depression within an individual’s lifetime, the durable imprinting of experience onto the microbiome may even contribute to inter-individual or trans-generational transfer of phenotypes (Callaghan, 2017; Elgart and Soen, 2018; Chu et al., 2019). While horizontal and vertical transmission of microbial elements have been demonstrated (Goodrich et al., 2016; Ferretti et al., 2018; Valles-Colomer et al., 2023), future research should characterise the specific imprints (such as relevant microbial pathways or microbe-host signalling molecules), and the extent to which natural transmission of parts of the imprinted-microbiota is sufficient to contribute to the phenotype in another individual.

## 6. Discussion

The bidirectional microbiota-gut-brain axis links stress to depression: the percept of stress shapes the microbiota *via* the efferent brain-gut axis, and the gut microbiota consequently modulates neural circuits *via* the afferent gut-brain axis.

On an optimistic note, while stress perception in the brain can lead to dysbiosis of the gut microbiota, the same efferent brain-gut conduit potentially also empowers the brain to sculpt the gut microbial community favourably. Indeed, mental states of tranquillity or mindfulness through regular meditation are linked to gut microbiota differences (Jia et al., 2020; Khine et al., 2020; Sun et al., 2023). Our own mind, by tapping into the innate brain-gut-microbiota axis, wields immense power over our gut microbiota (Figure 2). We also envisage that upcoming developments in elucidating such top-down host-microbe biology will transform science and society.

## Author contributions

The author confirms being the sole contributor of this work and has approved it for publication.

## Funding

This study was supported by Singapore National Medical Research Council (OFYIRG20nov-0044), Nanyang Technological University (020403-00001), and Agency for Science, Technology and Research (BMRC/IMCB/BBI).

## Conflict of interest

The author declares that the research was conducted in the absence of any commercial or financial relationships that could be construed as a potential conflict of interest.

## Publisher’s note

All claims expressed in this article are solely those of the authors and do not necessarily represent those of their affiliated organizations, or those of the publisher, the editors and the reviewers. Any product that may be evaluated in this article, or claim that may be made by its manufacturer, is not guaranteed or endorsed by the publisher.
